# Genetic Dissection of a Prevalent Plasmid-Encoded Conjugation System in *Lactococcus lactis*

**DOI:** 10.3389/fmicb.2021.680920

**Published:** 2021-05-28

**Authors:** Guillermo Ortiz Charneco, Philip Kelleher, Andrius Buivydas, Hugo Streekstra, Emiel Ver Loren van Themaat, Paul P. de Waal, Jennifer Mahony, Douwe van Sinderen

**Affiliations:** ^1^School of Microbiology and APC Microbiome Ireland, University College Cork, Cork, Ireland; ^2^DSM Biotechnology Center, Delft, Netherlands

**Keywords:** lactococci, starter culture, dairy fermentation, conjugative plasmid, recombineering, mutagenesis, horizontal transfer, co-mobilization

## Abstract

Plasmid pNP40, which was first identified nearly 40 years ago in *Lactococcus lactis* subsp. *lactis* biovar diacetylactis DRC3, encodes functions such as heavy metal-, bacteriophage-, and nisin-resistance, as well as plasmid transfer ability by conjugation. Here, we report an optimized conjugation protocol for this plasmid, yielding a transfer frequency that is approximately 4,000-fold higher than those previously reported in literature, while we also observed high-frequency plasmid co-mobilization. Individual mutations in 18 genes that encompass the presumed conjugation cluster of pNP40 were generated using ssDNA recombineering to evaluate the role of each gene in the conjugation process. A possible transcriptional repressor of this conjugation cluster, the product of the *tra*R gene, was identified in this manner. This mutational analysis, paired with bioinformatic predictions as based on sequence and structural similarities, allowed us to generate a preliminary model of the pNP40 conjugation machinery.

## Introduction

*Lactococcus lactis* and *Lactococcus cremoris* represent two Gram-positive, non-spore forming bacterial species, and members of the lactic acid bacteria (LAB), a group of micro-aerophilic coccoid and rod-shaped bacteria that produce lactic acid as the main product from hexose fermentation (Makarova et al., 2006; Ainsworth et al., 2014a; Li et al., 2021). *L. lactis* and *L. cremoris* are commonly used as a starter cultures in cheese, buttermilk, and quark production, with many technologically relevant traits being plasmid-encoded (Tarazanova et al., 2016), such as growth in milk (i.e., metabolism of lactose, citrate, and/or casein), resistance to environmental stresses and viruses (e.g., bacteriophages or heavy metals), and competitive advantages such as bacteriocin production (Fallico et al., 2012). Plasmids encoding these features may be transferable and/or mobilizable by conjugation, thus facilitating their rapid spread among bacteria within the same ecological/industrial niche (Mills et al., 2006). The generally recognized as safe (GRAS) status of LAB combined with their potential probiotic properties (Gurien et al., 2018), absence of endotoxins and inclusion bodies, and availability of a diverse selection of cloning and inducible expression vectors render LAB (bio)technologically interesting (Song et al., 2017). A relatively limited number of *L. cremoris* and *L. lactis* strains are currently used in large-scale fermentation processes, and this practice may have contributed to the somewhat narrow flavor diversity among dairy products and emergence of ubiquitous host-specific lytic bacteriophages (Coffey and Ross, 2002; Fallico et al., 2012; Li et al., 2021). This has prompted ongoing studies to identify and/or generate novel strains with improved phage-resistance, bacteriocin production or immunity, and/or flavor-associated properties.

Conjugation involves the transfer of genetic material via a conjugative apparatus from a donor to a recipient cell through direct pilus/adhesin-mediated cell-to-cell contact and the formation of a membrane-spanning channel through which the DNA is transported as a single stranded DNA (ssDNA) molecule (Kohler et al., 2019). The basic conjugation machinery appears to be conserved, typically being specified by a cluster of adjacent genes. Detailed information on the molecular mechanism and regulation of the conjugation process, particularly in Gram-positive bacteria, remains scarce though certain genetic functions can be identified based on their sequence conservation. Such genetic conjugation functions are generally referred to as transfer (*tra*) genes, which specify various activities such as nickases/relaxases, adhesins, and DNA transfer proteins (Harris and Silverman, 2004).

In both Gram-positive and Gram-negative bacteria, the conjugation process is strictly regulated to avoid fitness cost (Koraimann and Wagner, 2014), and the genes that form the conjugation cluster are either induced by signaling molecules (such as pheromones produced by plasmid-free recipient cells in conjugation mediated by the enterococcal plasmids pAD1 and pCF10) or are constitutively produced at low concentrations to reduce the metabolic burden, such as in the case of the *Bacillus subtilis* plasmid pLS20 (do Carmo de Freire Bastos et al., 1998; Bañuelos-Vazquez et al., 2017; Kohler et al., 2019; Meijer et al., 2021). Conjugation-related genes tend to be clustered together to form the so-called *tra* locus. The early assignment of these genes was based on an alphabetic system since many of the functions were unknown at that time (where the gene order was designated to be *tra*A, B, C, D, and E, etc.) (Curtiss, 1969). This nomenclature is problematic in more recently identified systems since the order of these genes and their encoded conjugation functions are not necessarily conserved (Kurenbach et al., 2002).

Plasmids that do not encompass a functional *tra* operon may be transferred by co-mobilization with conjugative plasmids, if they contain an origin of transfer (*ori*T) sequence and at least one mobilization gene: *mob*A, *mob*D (encoding nickases), *mob*B and *mob*C (encoding proteins that are thought to form a relaxosome with an associated nickase, either *mob*A or *mob*D) (O’Brien et al., 2015; Kelleher et al., 2019). Plasmids containing highly similar *ori*T sites can lack *mob* or *tra* genes but, when a conjugative plasmid is present together with a non-conjugative plasmid in the same donor strain, the relaxase from the former may recognize the *ori*T sequence within the non-conjugative plasmid, promoting transfer of either or both plasmids to a recipient cell (Francia et al., 2004). An example of this event is the co-mobilization of streptococcal plasmid pMV158 by pIP501 between strains of *Streptococcus pneumonia* (Grohmann et al., 2003). Therefore, conjugative plasmids without any known beneficial traits can still be used to mediate the transfer of non-conjugative plasmids with known beneficial characteristics.

The conjugative lactococcal plasmid pNP40, originally identified in *L. lactis* subsp. *lactis* biovar diacetylactis DRC3 (McKay and Baldwin, 1984), has been shown to encode various functions such as cold shock proteins, nisin-, cadmium-, and copper-resistance, as well as three distinct bacteriophage-resistance systems, AbiE, AbiF (Garvey et al., 1995), and LlaJI (O’Driscoll et al., 2004). While the presence of conjugative plasmids has been known in *L. lactis* and *L. cremoris* for some time, they have not been characterized in any detail, thus prompting a molecular analysis of the functionality of the conjugation-related genes of pNP40 while an optimized conjugation protocol is also described herein. Furthermore, pNP40-mediated plasmid co-mobilization has been described previously (Harrington and Hill, 1991), which prompted an evaluation of this phenomenon in *L. lactis* DRC3.

## Materials and Methods

### Bacterial Strains, Plasmids, and Growth Conditions

Lactococcal strains used in this study are listed in Table 1. Overnight cultures of lactococcal strains were grown at 30°C for 16 h in M17 + 0.5% (v/v) glucose (GM17) containing either nisin (2.5 μg/ml, for selection of *L. lactis* DRC3), streptomycin (500 μg/ml, to select for *L. cremoris* MG1614) (Gasson, 1983), chloramphenicol (5 μg/ml, to select for *L. cremoris* NZ9000 pJP005), tetracycline (10 μg/ml, to select strains harboring pPTPi) and erythromycin (5 μg/ml, for pNZ44E-containing strains). Electrocompetent cells of *L. cremoris* were prepared as described previously (Holo and Nes, 1989).

**TABLE 1:** Lactococcal strains used in this paper: *Lactococcus lactis* DRC3 (McKay and Baldwin, 1984) was employed as donor in the mating assays, whereas *Lactococcus cremoris* MG1614 (Gasson, 1983) and *L. cremoris* NZ9000 harboring pJP005 were used as recipients in this study.

Strain	Plasmids present in the strain	Characteristics
**DRC3**	pDRC3-A pDRC3-B (pNP40) pDRC3-C pDRC3-D pDRC3-E pDRC3-F pDRC3-G	Donor strain with the conjugative plasmid pNP40, providing nisin resistance encoded by *nisR*
**MG1614**	–	Plasmid-free strain, resistant to streptomycin and main recipient in this study
**NZ9000**	pJP005	Plasmid encoding *RecT* and chloramphenicol resistance

*Although the strain *L. cremoris* NZ9000 is plasmid free, for recombineering purposes the strain used in this study harbors plasmid pJP005 (Van Pijkeren and Britton, 2012).*

### Mating Experiments

Three distinct conjugation strategies were assessed (Supplementary Figure 1): (i) Solid mating, (ii) Filter mating, and (iii) Spread solid mating. For all three strategies, 10 ml of an overnight culture of donor and recipient was obtained after growth at 30°C in GM17 to which, where relevant, antibiotics had been added. For the solid and filter mating approaches, these overnight cultures were diluted 1/1,000 in 10 ml fresh GM17 with antibiotics where relevant and grown to an OD_600 *nm*_ of 0.7. For the spread solid mating approach, the overnight culture was used directly for the next step, being the same for all strategies: cells from 10 ml of culture were harvested by centrifugation for 15 min at 3,000×*g* and resuspended in 5 ml of antibiotic-free GM17. Donor and recipient cells were then mixed in a 1:1 ratio to a final volume of 2 ml and the mixture was centrifuged for 5 min at 3,000×*g* and, depending on the strategy to be followed: (i) (for solid mating) the pellet was resuspended in 300 μl of 5% reconstituted skim milk (RSM) supplemented with 2% glucose and spotted on the center of 5% RSM, 2% glucose agar plates, allowing the mixture to dry in the center of the plate, without spreading to the edges; (ii) (for filter mating) the pellet was resuspended in 5 ml of GM17 and vacuum-filtered onto sterile 13 mm nitro-cellulose filters (HA; Millipore), after which filters were placed cell-side-up onto 5% RSM, 2% glucose agar plates; (iii) (for spread solid mating) the pellet was resuspended in 300 μl of 5% RSM, 2% glucose and evenly spread on 5% RSM, 2% glucose agar plates. Plates for all three conditions were then incubated overnight at 30°C, the cells were scraped from the plate and resuspended in 4 ml of Ringer’s solution and 0.1 ml volumes of serial dilutions were plated onto GM17 agar plates. The agar plates were selective for recipient cells (supplemented with either streptomycin or chloramphenicol, for *L. cremoris* MG1614 or NZ9000 pJP005, respectively) or transconjugants (supplemented with nisin for selection of pNP40 and streptomycin or chloramphenicol, depending on the recipient strain used). The presence of pNP40 in *L. cremoris* MG1614 cells was further verified by using pNP40- and MG1614-specific primers (Supplementary Table 2), confirming conjugative transfer of pNP40 to the recipient strain.

Liquid mating was also evaluated in this study in which overnight cultures of the donor and recipient strains were inoculated together in a 1:1 ratio (100 μl of each) into GM17 broth (10 ml) and incubated at 30°C. When the OD_600 *nm*_ of this mixed culture reached 0.8, the mixture was then serially diluted and plated on agar plates that were selective either for the recipient (supplemented with streptomycin for the selection of the recipient *L. cremoris* MG1614) or the transconjugant cells (supplemented with nisin for pNP40 selection, and streptomycin).

The conjugation frequency was calculated based on the number of recipients that had received plasmid pNP40 as a percentage of the overall recipient population, using the following formula:

$$\text{Frequency}\text{of}\text{conjugation}\left(\%\right)=\left(\frac{\frac{c\,f\,u}{m\,l}\,\left(\text{transconjugants}\right)}{\frac{c\,f\,u}{m\,l}\,\left(\text{recipients}\right)}\right)\times 100$$

### Recombineering in *L. lactis*

Oligonucleotides for recombineering are listed in Supplementary Table 2. The primers were designed based on a previously described and optimized approach for ssDNA recombineering (Van Pijkeren and Britton, 2012; Stockdale et al., 2013; Ainsworth et al., 2014b). Following conjugation between *L. lactis* DRC3 and *L. cremoris* NZ9000 pJP005, a strain capable of facilitating both conjugation and recombineering was produced, designated NZ9000 pNP40, pJP005. Electrocompetent cells harboring pJP005 and pNP40 were prepared as previously described (Holo and Nes, 1989), and 45 μl of cells and 400 μg of oligonucleotide were electroporated at 2,000 V, 25 μF, and 200 Ω. Following electroporation, 1 ml of GM17 was added and bacteria were recovered for 1.5 h at 30°C, followed by serial dilution and plating on GM17 agar plates containing 2.5 μg/ml nisin.

For nisin-mediated *rec*T induction, 15 μl of filtered supernatant of an overnight culture of *L. cremoris* NZ9700 (a nisin-producing strain) per ml of to be induced culture was used, and this culture was incubated at 30°C for 2 h. Competent cells of *L. cremoris* NZ9000 harboring pJP005 and pNP40 were transformed with 400 μg oligonucleotide and, after recovery, serial dilutions were plated on GM17 agar plates containing nisin. Eighteen genes encompassing the predicted conjugation gene cluster of pNP40 were selected as individual targets for recombineering. For the inactivation of each gene a 90-mer oligonucleotide was designed (following primer design recommendations as outlined by Van Pijkeren and Britton, 2012) such that, upon incorporation, they would introduce two in-frame stop codons, thereby terminating translation, and an EcoRI recognition sequence (mutant nomenclature *tra*_*pNP40*_::Ter) (Supplementary Table 2). The so-called mismatch amplification mutation analysis-PCR (MAMA-PCR) method was used to screen individual colonies for the presence of mutants (Cha et al., 1992; Qiang et al., 2002; Van Pijkeren and Britton, 2012). Once a mutant genotype was identified, the remaining portion of the “positive” colony was streaked on a GM17 agar plate supplemented with nisin in order to purify the mutant from possible unmutated/wildtype (WT) cells. This procedure was repeated until a pure mutant was obtained, and with this pure culture, colony PCR was performed using two oligonucleotides (Supplementary Table 2) that bind ∼500 bp upstream and downstream of the intended mutated sequence, yielding a ∼1 kb fragment, which was sequenced by Sanger sequencing (Eurofins, Ebersberg, Germany) to validate that the expected mutation was present in the pNP40-derived plasmid. The obtained mutants were next assessed for conjugation, employing the spread solid mating approach.

### Complementation and Overexpression

Mutants were genetically complemented to ascertain if the phenotype of reduced conjugation frequency (compared to the WT strain) could be restored. Genes, which upon mutation had been shown to exhibit a decrease in conjugation frequency, were individually cloned into the low copy plasmid pPTPi, under the control of the nisin-inducible promoter of pNZ8020 (O’Driscoll et al., 2004), incorporating a native or artificial Shine-Dalgarno sequence. Induction of this promoter was achieved by the inclusion of nisin (5 ng/ml) in the growth media. These pPTPi-derived constructs were individually transformed into the relevant NZ9000 pNP40, and pJP005 mutated derivative. Agar plates were selective for both pNP40 and pPTPi through the incorporation of nisin (for pNP40 selection) and tetracycline (for pPTPi selection).

Genes from the pNP40 conjugation cluster for which inactivation did not appear to affect conjugation frequency (or where the mutation was shown to increase conjugation), were individually cloned into the high copy, expression plasmid pNZ44E, an erythromycin-resistant derivative of pNZ44, with a constitutive P44 promoter (Draper et al., 2009). Transformants were selected on agar plates containing nisin and erythromycin (selection for pNP40 and pNZ44E, respectively). The resulting strains were used as donors for conjugation using the spread solid mating protocol to test if their overexpression affect conjugation frequency, and in one case employing liquid mating, which represents a condition resulting in undetectable levels of conjugation in the case of the wild type situation.

### Isolation of Genomic DNA

Genomic DNA from *L. lactis* DRC3 was isolated from bacteria harvested in the exponential growth phase using Nucleobond^®^ AXG columns and the Nucleobond^®^ buffer set III (Macherey-Nagel Gmbh, Düren, Germany). The protocol used was taken from “Genomic DNA and RNA purification–User manual” of July 2015, revision 08 (Macherey-Nagel GMbh, Düren, Germany) following the “Protocol for Nucleobond^®^ AXG Columns and Nucleobond^®^ Buffer Set III” for “Isolation of genomic DNA from bacteria” with the following modifications. For cell disruption, 30 mg/ml of lysozyme was added, and incubation time was set to 16 h. Incubation at 50°C after the addition of Buffer G4 was set to 1 h, and in the binding step 8 ml of Buffer N2 was used. In the final precipitation step, the obtained pellet was dissolved in 10 mM Tris Buffer (pH 8.00) and incubated at 55°C for 1 h before final storage.

### Genome Sequencing, Assembly, and Annotation

Sequencing was performed utilizing a combined SMRT sequencing and Illumina approach on a Pacific Biosciences RS II sequencing platform (executed by GATC Biotech Ltd., Germany) and an Illumina MiSeq platform (executed by GenProbio s.r.l., Parma, Italy). *De novo* assemblies were performed on the Pacific Biosciences SMRTPortal analysis platform (version 2.3.1), utilizing the RS_HGAP_Assembly.2 protocol. Hybrid assemblies were performed utilizing the Unicycler hybrid assembly pipeline (Wick et al., 2017). Remaining low quality regions and sequencing conflicts were resolved by primer walking and Sanger sequencing of PCR products (performed by Eurofins MWG Operon, Germany). Open Reading Frame (ORF) prediction was performed with Prodigal v2.5 prediction software (Hyatt et al., 2010) and confirmed using BLASTX v2.2.26 alignments (Altschul et al., 1990). ORFs were automatically annotated using BLASTP v2.2.26 (Altschul et al., 1990) analysis against the non-redundant protein databases curated by the National Centre for Biotechnology Information (NCBI). Artemis v18 genome browser and annotation tool was used to manually curate ORFs and for the combination and inspection of ORF results. Final ORF annotations were refined where necessary using alternative databases; Pfam (Bateman et al., 2004), HHpred (Söding et al., 2005), and Uniprot/EMBL.

### pNP40-Mediated Plasmid Co-mobilization

Eight sets of primers of were designed (Supplementary Table 2), targeting unique sequences within each of the seven plasmids present in *L. lactis* DRC3 (including pNP40) and the *L. lactis* DRC3 genome. The amplicons of each primer pair were distinct allowing identification of the associated plasmid (Supplementary Table 3). These primers were designed to produce different size products: DRC3-A (1,483 bps product size), DRC3-B (1,227 bps), DRC3-C (1,000 bps), DRC3 chromosome (852 bps), DRC3-D (723 bps), DRC3-E (579 bps), DRC3-F (478 bps), and DRC3-G (306 bps). A multiplex polymerase chain reaction (mPCR) approach was then adopted to assess plasmid co-mobilization (Markoulatos et al., 2002). Primers were designed with similar reaction kinetics and the mPCR was performed as follows: single colonies were picked from the transconjugant plates and added to a total reaction volume of 50 μL, using Phusion Green High-Fidelity DNA Polymerase (2 U/μL) (Thermo Fisher Scientific, Waltham, MA, United States). An initial 95°C denaturation step was performed for 10 min, after which a 35-cycle PCR procedure was applied (1 min denaturation at 95°C, 30 s annealing at 50°C and 1 min extension at 72°C). A final extension step at 72°C for 7 min was performed, after which samples were stored until further use.

Conjugation was performed under optimized conditions, following the spread solid mating protocol (as detailed above), between *L. lactis* strain DRC3 and *L. cremoris* MG1614, and the colonies present in the donor (positive control, supplemented with nisin), recipient (negative control, with streptomycin added) and transconjugant-specific plates (supplemented with nisin and streptomycin) were randomly selected for PCR screening using the mPCR method.

### Comparative Genomics

All sequence comparisons at protein level were performed via all-against-all, bi-directional BLAST alignments (Altschul et al., 1990). An alignment cut-off value of E-value 0.0001, >30% amino acid identity across 80% of the sequence was used. For the analysis and clustering of these results, the Markov Clustering Algorithm (MCL) was implemented in the mclblastline pipeline v12-0678 (Enright et al., 2002).

### Functional Analysis of the pNP40 Conjugation System

All conjugative gene and protein sequences from the pNP40 conjugation gene cluster were compared using BLAST. Furthermore, TMHMM v.2.0 software was used for the prediction of transmembrane helices in these proteins using the hidden Markov model (HMM). Additionally, HHpred analysis was performed, which provided remote protein homology detection and structure prediction with pairwise comparison of profile HMM (Söding et al., 2005; Zimmermann et al., 2018), while Pfam (El-Gebali et al., 2019) was used to identify/confirm the presence of functional domains.

### Statistical Data Analysis

All experiments were performed in triplicate. Data presented are means ± standard deviation (SD). Results were analyzed using the SigmaPlot 11.0 statistical package (SPSS). A value of *P* < 0.05 was considered significant and is represented in the graphs with a single asterisk “^∗^”, while a value of *P* of ≤0.001 was considered highly significant and is represented by two asterisks “^∗∗^” in the graphs.

## Results

### Comparative Genomics

Three plasmid-encoded conjugation systems have been described among lactococci to date, i.e., those specified by plasmids pMRC01, pAF22, and pNP40, which all harbor gene clusters encoding proteins with similarity to components of known conjugation systems (Harrington and Hill, 1991; Coakley et al., 1997; O’Driscoll et al., 2006; Fallico et al., 2012). We aimed to assess the presence and diversity of these previously described lactococcal conjugation-associated clusters among publicly available plasmid sequences (Kelleher et al., 2019). Comparative analysis was performed via all-against-all, bi-directional BLASTP alignment, and clustering implemented in the MCL pipeline (Altschul et al., 1990; Enright et al., 2002), using 222 lactococcal plasmids currently available in the NCBI database (October 2020).

**FIGURE 1:**
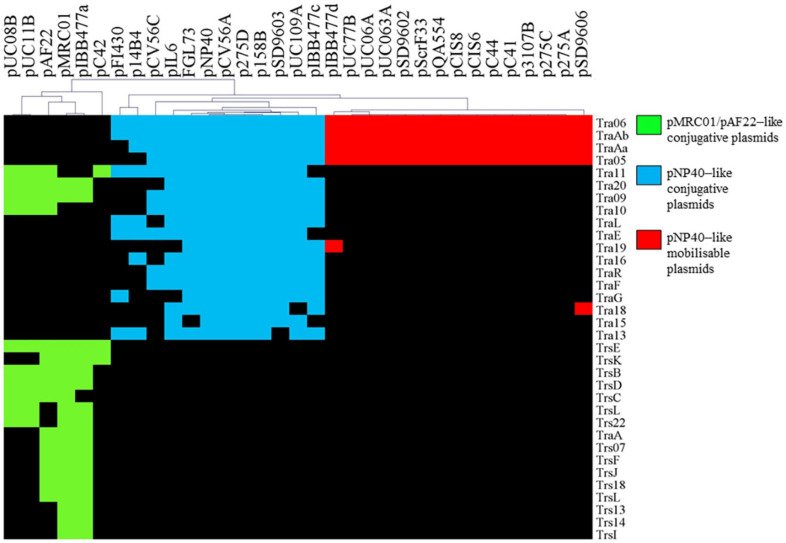
Hierarchal clustering analysis representing the presence/absence of gene families from conjugation clusters of pAF22, pMRC01, and pNP40-like plasmids. Gene clusters are indicated based on the hierarchical tree, top. Color indications refer to the three different conjugative/mobilizable plasmid types identified. Gene names were assigned based on the *tra* operon of pNP40 (this study) and pMRC01 (publicly available data).

Markov Clustering Algorithm analysis of predicted pMRC01-, pAF22-, and pNP40-like conjugation-related genes across the current NCBI database of lactococcal plasmids resulted in a gene presence/absence matrix displaying three groupings of plasmids (Figure 1). Gene names were assigned based on the *tra* operon of pNP40 (as assigned in the current work, see below) and the conjugation operon from the publicly available sequence of pMRC01 (Dougherty et al., 1998). When the homology of the genes was significantly similar between groups, they were assigned the nomenclature of pNP40 (i.e., *tra*11, *tra*20, tra09, and *tra*10). Among the 222 lactococcal plasmids analyzed, 33 contain conjugation-related genes homologous to those encoded by pAF22, pMRC01, or pNP40. In most cases, these conjugation genes tend to be clustered together, forming what is typically called the *tra* locus (Curtiss, 1969). Among the 33 plasmids harboring conjugation-associated genes, six (pUC08B, pUC11B, pAF22, pMRC01, pIBB477a, and pC42) presented a partial or complete set of conjugation-related genes with significant homologies between them, that were for this reason categorized as pMRC01/pAF22-like conjugative plasmids. Twelve of the plasmids (pFI430, p14B4, pCV56C, pIL6, pGGL73, pSD9603, pNP40, pCV56A, p275, p158B, pUC109A, and pIBB477c) had partial or complete sets of conjugation-related genes with significant homology to each other, being grouped here as pNP40-like conjugative plasmids. Finally, a third group, comprised of fifteen plasmids (pIBB477d, pUC77B, pUC06A, pUC063A, pSD9602, pScrF33, PQA554, pCIS8, pCIS6, pC44, pC41, p3107B, p275C, p275A, and pSD9606), was classified based on a highly conserved set of four pNP40-associated conjugation-related genes, *tra*06, *tra*05, *tra*Aa, and *tra*Ab. Although the precise role of *tra*06 and *tra*05 in the conjugation process has not yet been determined, *tra*Aa and *tra*Ab are predicted to encode mobilization proteins, a MobD-like relaxase (encoded by *tra*Ab) and a MobC-like accessory factor (encoded by *tra*Aa). Since these four genes are highly conserved among these fifteen plasmids, they were classified as pNP40-like mobilizable plasmids, as they contain mobilization-associated genes homologous to those present on pNP40, yet lack other genes typically required for self-transfer. These findings imply that these fifteen plasmids are not conjugable by themselves yet may be mobilizable. A closer look into the published sequence of these fifteen plasmids showed that they do not appear to share any common functions, while a multiple sequence alignment of the nucleotide sequences of the fifteen plasmids (Supplementary Figure 4) confirmed that they are not related to each other outside their common conjugation/mobilization-related genes.

Based on this comparative analysis, pNP40-like conjugation systems would appear to be the most prevalent among lactococcal plasmids annotated in public databases, thus prompting further characterization of the pNP40 conjugation system.

### High Frequency Conjugation in *L. lactis*

The conjugal capacity of pNP40 has previously been demonstrated, although conjugation frequencies were reported to be rather low, with pNP40 transconjugants obtained at a reported frequency of ∼3–5 × 10^–4^% (transconjugants per donor cell) (Harrington and Hill, 1991; Trotter et al., 2001). This prompted an evaluation of the conjugation protocols/parameters to improve and optimize the conjugation frequency of pNP40 as a model for related conjugative lactococcal plasmids.

**FIGURE 2:**
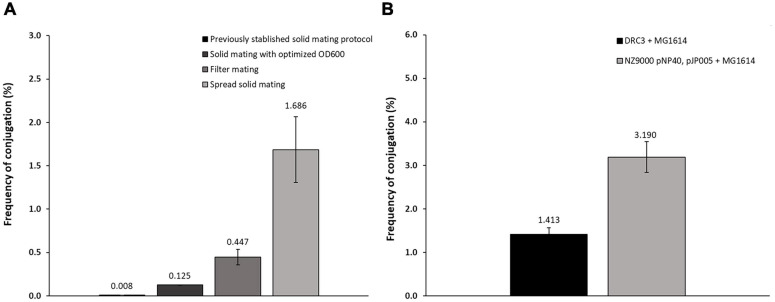
**(A)** Conjugation frequencies for the different protocols employed. Following a previously established protocol (Trotter et al., 2001), conjugation frequencies achieved were around 0.008%. Varying the OD_600nm_ at which the cultures were centrifuged together (0.7) allowed an increase to 0.125%. Using the filter mating protocol, the frequency increased to around 0.447%, while the spread solid mating protocol further improved conjugation to 1.686%. **(B)** Conjugation frequency comparison of: conjugation control (DRC3 and MG1614) and conjugation between NZ9000 pNP40, pJP005, and MG1614, showing in this “two-step conjugation” a substantial increase in conjugation frequency when compared to the control.

Based on previously described protocols (Harrington and Hill, 1991; Trotter et al., 2001), optimization of pNP40 conjugation efficacy was undertaken employing the donor *L. lactis* DRC3 and the recipient strain *L. cremoris* MG1614, using three distinct approaches (designated here as solid mating, filter mating, and spread solid mating) as described in the “Materials and Methods” section. Conjugation frequencies ranged from 0.008 to 1.686% (percentage of transconjugants per recipient cell) (Figure 2A), with the lowest frequency obtained following a previously established protocol (Trotter et al., 2001) and the highest conjugation frequency observed when the donor and recipient cell mixtures were evenly spread onto 5% RSM, 2% glucose agar plates, when performing the spread solid mating protocol (see “Materials and Methods”). In the filter mating approach, conjugation frequencies were ∼0.5%, which meant a fourfold increase when compared to the non-optimized solid mating protocol, which yielded a conjugation frequency of 0.125%. In contrast, no detectable conjugation was observed when employing the liquid mating protocol, as no colonies were observed in the transconjugant plates with an associated limit of detection of <1 × 10^–5^%.

To perform mutagenesis studies, pNP40 was transferred by conjugation to *L. cremoris* NZ9000 pJP005, a strain which allows mutagenesis by so-called recombineering (Van Pijkeren and Britton, 2012), resulting in the transconjugant strain *L. cremoris* NZ9000 pNP40, pJP005. This transconjugant strain was then assessed for its use as a donor of pNP40 (or its mutated derivatives) to the recipient strain *L. cremoris* MG1614. Indeed, employing *L. cremoris* NZ9000 pNP40, pJP005, and *L. cremoris* MG1614 as a donor/recipient combination allowed highly efficient pNP40 conjugation at a frequency of approximately 3.2% (Figure 2B), which is more than twice the frequency obtained when compared to *L. lactis* DRC3-*L. cremoris* MG1614 donor-recipient combination.

The presence of pNP40 in *L. cremoris* MG1614 cells was verified by both a nisin resistance phenotype and a PCR-based genetic confirmation (see “Materials and Methods”). This conjugation optimization resulted in a more than 200-fold increase in conjugation frequency above some of the other methods tested in this study, and an approximate 4,000-fold increase when compared to findings reported in previous studies using this plasmid (Supplementary Table 5) (Harrington and Hill, 1991; Trotter et al., 2001).

### Predictive Functional Analysis of the pNP40 Conjugation Operon

**FIGURE 3:**
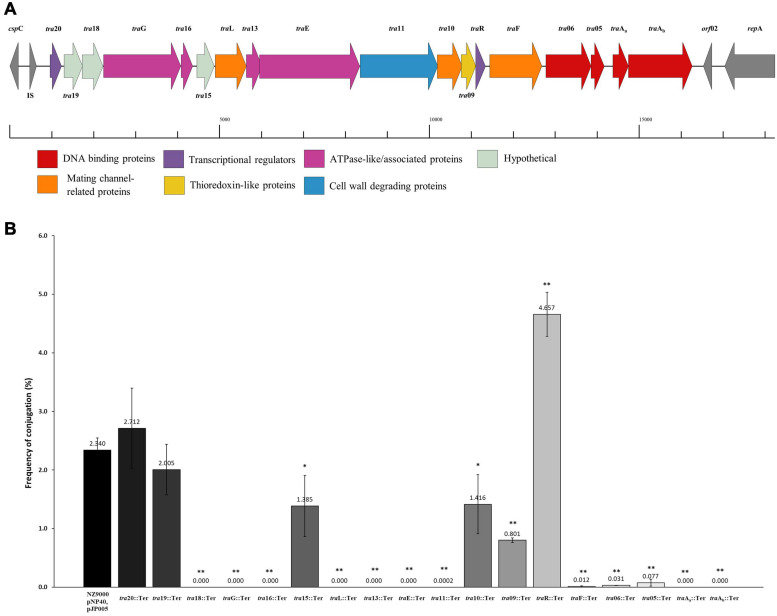
Functional and mutational analysis of the pNP40 conjugation cluster. **(A)** pNP40 conjugation cluster, showing the organization of the 19 genes that compose it and their assigned functions. **(B)** Conjugation frequencies after mutating each individual gene from the pNP40 conjugation cluster. All conjugations were done using as donor the recombinant strain (NZ9000 pNP40, pJP005, and with the individual mutation) and MG1614 as the recipient. Conjugation between a wild-type donor and MG1614 was used as a control. A p-value < 0.05 was considered significant and is represented with a single asterisk “^∗^”, while a p-value ≤ 0.001 was considered very significant and is represented by two asterisks “^∗∗^”. Data are the mean of three replicates ± standard deviation.

Based on a previous study (O’Driscoll et al., 2006), a conjugation-related cluster encompassing eighteen genes in pNP40 had been identified and selected for functional analysis. This conjugation cluster is delimited at its 5’-end by a cold shock-related gene (*csp*C) and one insertion sequence element, and a replication-associated gene (*rep*A) at its 3’-end (Figure 3A). A preliminary comparison of DNA and protein sequences of the pNP40 conjugation-related genes and their associated products using an array of sequence and structural similarity search tools to identify functional domains are summarized in Table 2.

**TABLE 2:** Functional analysis of the genes from the pNP40 conjugation cluster, based on amino-acid and protein sequence comparison using BLAST and Pfam v32.0, TMHMM for the prediction of transmembrane helices in proteins and HHpred to assign function based on structure prediction.

Gene	BLAST	Pfam	HHpred	TMHMM (transmembrane domain)
***tra20***	Hypothetical	Insignificant matches	Conjugation dsDNA binding/*tra*N similarity	No
***tra19***	Hypothetical	No matches	Uncharacterized	Yes (three domains)
***tra18***	Hypothetical	Insignificant matches	Intramembrane protease/Hydrolase	No
***traG***	*tra*G family protein, type IV secretory system	Type IV secretory system	Coupling protein/VirB4-like/ATPase	Yes (two domains)
***tra16***	Potassium-transporting ATPase A chain	Insignificant matches	Transmembrane cytochrome	No
***tra15***	Hypothetical	Insignificant matches	Uncharacterized	Yes (four domains)
***traL***	TrbL homologue	Mating pair stabilization pore-forming protein	Uncharacterized	Yes (five domains)
***tra13***	PrgI family protein, type IV secretion system	PrgI family protein	ESX-3 secretion system protein	Yes (two domains)
***traE***	Conjugation protein *tra*E	Insignificant matches	Type IV secretion system, ATPase	No
***tra11***	*traG*-like protein	CHAP domain/Amidase	Cell wall/peptidoglycan hydrolase	Yes (one domain)
***tra10***	Hypothetical	Insignificant matches	Membrane protein, *tra*M similarity/unknown	Yes (one domain)
***tra09***	Thioredoxin	Thioredoxin	Thioredoxin-like fold, transferase	No
***traR***	MerR family transcriptional regulator	Histone acetyltransferase	DNA binding protein/transcriptional regulator	No
***traF***	*tra*F conjugal protein	Insignificant matches	Membrane protein	Yes (one domain)
***tra06***	ImmA/IrrE family metallo-endopeptidase	Metallopeptidase domain	DNA binding protein	No
***tra05***	Hypothetical	Insignificant matches	Histone-fold, DNA binding	No
***traA*_*a*_**	*mob*C relaxosome protein	*mob*C	Mobilization protein *mob*C, accessory factor	No
***traA*_*b*_**	Relaxase/nuclease *mob*D	Relaxase	Mobilization protein A, nickase/DNA relaxase	No

Gene products were divided into one of seven groups based on their putative function, as shown in Figure 3A. Gene names were assigned based on either their position in the pDRC3-B plasmid, their similarities with known conjugation-related genes (*tra*G, *tra*L, *tra*E, and *tra*F) or their functionality (*tra*R, *tra*Aa, and *tra*Ab). The first group is composed of gene products of *tra*06, *tra*05, *tra*Aa, and *tra*Ab were identified as DNA binding proteins. Among these, TraAb displays similarity to the nickase-relaxase family of proteins, particularly MobA, which is known to introduce a nick at the *ori*T to initiate single-stranded plasmid DNA transmission from donor to recipient. TraAa is similar to the mobilization protein MobC, suggestive of a role as an accessory factor for the relaxase, both of which are essential for conjugation (Smith and Thomas, 2004). The second group comprises predicted transcriptional regulators of the pNP40 conjugation cluster, encoded by *tra*R and *tra*20. Both TraR and Tra20 exhibit similarities to the MerR family of transcriptional regulators, and particularly Tra20 displays sequence similarity to TraN, reported to act as a repressor of the pIP501-encoded conjugation cluster (Grohmann et al., 2016). These findings suggest that the products encoded by these genes are involved in controlling the expression of the pNP40 conjugation machinery. Genes *tra*G, *tra*E, *tra*13, and *tra*16 form the third group of genes, encoding predicted ATPase or ATPase-associated proteins. Particularly, TraG and the C-terminus of the amino acid sequence of TraE return a 100% probability (using a HHPred search) to belong with the family of TraG-like coupling proteins, which are responsible for recruiting the relaxosome for transport by the conjugation apparatus (Cabezón et al., 1997). These latter proteins also exhibit similarity to the VirB4-like family of ATPases from the *Agrobacterium tumefaciens* conjugative transfer machinery, VirB/D4 type IV secretion system (T4SS) (Atmakuri et al., 2004). The *tra13* gene is located upstream of the gene encoding the presumed equivalent of the VirB4-like ATPase, TraE. Tra13 is related to the PrgI family of proteins, which are small proteins with two or three transmembrane domains, whose encoding genes are located directly upstream of the *vir*B4-like genes, and which are postulated to functionally interact with VirB4-like ATPases (Alvarez-Martinez and Christie, 2009; Wallden et al., 2010). For this reason, Tra13 was categorized as an ATPase-associated protein, although its specific role in conjugation is still unknown. The fourth group consists of gene *tra*11. The product of *tra*11 contains a glucosaminidase and CHAP domains, both involved in cell-wall degrading activity (Bateman and Rawlings, 2003). The presence of a transmembrane domain suggests that this protein is membrane-bound, and this protein was thus predicted to be a membrane-associated protein with peptidoglycan-degrading activity. Genes *tra*L, *tra*10, and *tra*F comprise the fifth group, the mating channel proteins, which form the actual transport channel required for transfer of the ssDNA across the membrane. The sixth group is composed of a single gene, *tra*09, encoding a thioredoxin-like protein with an as yet unknown function in pNP40, although these proteins are known to promote the folding and maintenance of disulfide bond-containing proteins in the periplasm via a redox system (Hemmis and Schildbach, 2013).

### Mutagenesis of Individual Genes of the Conjugation Cluster of pNP40

A systematic mutational analysis of the genes from the pNP40 conjugation gene cluster was undertaken in this study to validate the predicted functions in the conjugation process. To achieve this, non-sense mutations were incorporated in each of the 18 genes that constitute the predicted pNP40 conjugation or *tra* cluster. Following the generation of these pNP40-derivatives, the corresponding recombinant strains were used individually as donors for conjugation with *L. cremoris* MG1614 (the recipient strain) and the impact of each mutation on conjugation frequency was assessed (Figure 3B). All cases were compared to a control, where unmutated NZ9000 pNP40, and pJP005 was used as the donor.

Based on this analysis, different levels of impact on the conjugative ability of the mutants were observed (Table 3). This ranged from apparently no impact (i.e., mutations in *tra*20 and *tra*19), mild impact with <10-fold decrease in conjugation frequency (i.e., mutations in *tra*15, *tra*10, and *tra*09), a major impact (up to 10,000-fold decrease, as observed for *tra*F, *tra*06, *tra*05, and *tra*11) and those that were deemed to be essential for conjugation (as was observed for mutations in *tra*18, *tra*G, *tra*16, *tra*L, *tra*13, *tra*E, *tra*Aa, and *tra*Ab). Interestingly, the *tra*R mutant displayed >twofold increased conjugation in comparison to the control.

**TABLE 3:** Classification of the genes from the pNP40 conjugation cluster based on their individual effect in conjugation frequency: >twofold increased conjugation, no impact in conjugation frequency; modest impact, <10-fold decrease; major impact, up to 10,000-fold-decrease; essential for conjugation.

Impact in conjugation	Genes
**Increased conjugation**	*tra*R
**No impact**	*tra*20 and *tra*19
**Modest impact**	*tra*15, *tra*10, and *tra*09
**Major impact**	*tra*11, *tra*F, *tra*06, and *tra*05
**Essential**	*tra*18, *tra*G, *tra*16, *tra*L, *tra*13, *tra*E, *tra*A_a_, and *tra*A_b_

### Complementation and Overexpression

To confirm if the reduction of conjugation efficiency in certain generated mutants was due to the incorporation of a non-sense mutation or through polar effects on downstream genes, complementation analysis was undertaken. Complementation analysis of mutants (summarized in Table 4) exhibiting a reduced or abolished conjugation ability phenotype caused complete restoration of conjugation frequencies (ranging from 1.8 to 2.1%) for all but two genes. Reintroduction of intact copies of genes *tra*13 and *tra*E into the mutant derivatives did not completely restore conjugation to wild type levels, but a restoration to approx. 0.5% was observed, representing a significant (*P* < 0.05) and obvious increase in conjugation frequency.

**TABLE 4:** Complementation and overexpression conjugation results.

Donor strains (*L. cremoris* NZ9000)	Conjugation frequency (%)	Donor strains (*L. cremoris* NZ9000)	Conjugation frequency (%)
*tra18*_*pNP40*_::Ter	0.000	*tra09*_*pNP40*_::Ter	0.801
*tra18*_*pNP40*_::Ter pPTPi::*tra18*	1.854	*tra09*_*pNP40*_::Ter pPTPi::*tra09*	2.188
*traG*_*pNP40*_::Ter	0.000	*traF*_*pNP40*_::Ter	0.012
*traG*_*pNP40*_::Ter pPTPi::*traG*	1.798	*traF*_*pNP40*_::Ter pPTPi::*traF*	2.124
*tra16*_*pNP40*_::Ter	0.000	*tra06*_*pNP40*_::Ter	0.031
*tra16*_*pNP40*_::Ter pPTPi::*tra16*	2.069	*tra06*_*pNP40*_::Ter pPTPi::*tra06*	1.995
*tra15*_*pNP40*_::Ter	1.385	*tra05*_*pNP40*_::Ter	0.077
*tra15*_*pNP40*_::Ter pPTPi-*tra15*	1.804	*tra05*_*pNP40*_::Ter pPTPi::*tra05*	2.164
*traL*_*pNP40*_::Ter	0.000	*traA*_*a*__–pNP40_::Ter	0.000
*traL*_*pNP40*_::Ter pPTPi::*traL*	1.891	*traA*_*a*__–pNP40_::Ter pPTPi::*traA*_*a*_	2.136
*tra13*_*pNP40*_::Ter	0.000	*traA*_*b*__–pNP40_::Ter	0.000
*tra13*_*pNP40*_::Ter pPTPi::*tra13*	0.486	*traA*_*b*__–pNP40_::Ter pPTPi::*traA*_*b*_	2.004
*traE*_*pNP40*_::Ter	0.000	*tra20*_*pNP40*_::Ter	2.712
*traE*_*pNP40*_::Ter pPTPi::*traE*	0.511	NZ9000_*pNP40*_ pNZ44E::*tra20*	2.264
*tra11*_*pNP40*_::Ter	0.000	*tra19*_*pNP40*_::Ter	2.005
*tra11*_*pNP40*_::Ter pPTPi::*tra11*	2.022	NZ9000_*pNP40*_ pNZ44E::*tra19*	2.164
*tra10*_*pNP40*_::Ter	1.416	*traR*_*pNP40*_::Ter	4.657
*tra10*_*pNP40*_::Ter pPTPi::*tra10*	1.844	NZ9000_*pNP40*_ pNZ44E::*traR*	0.192

*In all cases, *L. cremoris* MG1614 was used as the recipient strain. Results correspond to conjugation using either the mutated (*tra*_*pNP40*_::Ter), the complemented (*tra*_*pNP40*_::Ter pPTPi::*tra*) or the overexpressed (NZ9000_*pNP40*_ pNZ44E::*tra*) strains as donors. The spread solid mating protocol was employed for all cases.*

As demonstrated above, mutation of certain genes did not appear to affect (genes *tra*20 and *tra*19) or even increased (gene *tra*R) the corresponding conjugation frequency when compared to the wild type situation. To further assess if these genes play a role in conjugation regulation, they were overexpressed in unmutated NZ9000 pNP40 strains to discern if these gene products, when assumed to be present in higher concentrations (compared to the wild type situation), had an impact on conjugation frequency. High constitutive transcription of *tra20*, *tra19*, and *traR* was achieved by cloning these genes individually into plasmid pNZ44E. These constructs were then transformed into NZ9000 pNP40 strains, and the generated strains were used as donors for conjugation experiments (Table 4). When genes *tra*20 and *tra*19 were overexpressed, no significant difference was observed in conjugation frequencies (∼2%), whereas *tra*R overexpression yielded significantly (*P* < 0.05) reduced conjugation frequencies (∼0.2%). This decrease in conjugation frequency, paired with a conjugation increase when the same gene was inactivated, suggests that it plays a role in regulating expression or function of the pNP40 conjugation machinery.

As mentioned above, conjugation with the mutant strains was performed under optimized conditions (i.e., employing spread solid mating protocol). Lactococcal strains carrying pNP40 in which *tra*20, *tra*19, or *tra*R had been inactivated were selected, based on their conjugation frequencies and functional analysis, for a subsequent conjugation experiment under suboptimal conjugation conditions (i.e., non-optimized solid mating and liquid mating, using *L. cremoris* MG1614 as a recipient), to determine if their conjugation frequencies would be even more significantly different from the control. The results from these conjugation experiments are summarized in Table 5. When using NZ9000 *tra20*_*pNP40*_::Ter and *tra19*_*pNP40*_::Ter strains as donors, following the non-optimal solid mating protocol, conjugation frequencies were reduced to 7.02 × 10^–3^ and 6.4 × 10^–3^%, respectively, which is not significantly different from the ∼8 × 10^–3^% obtained in the positive control (in which the unmodified NZ9000 pNP40 was used as a donor). In the case of the liquid mating protocol, no conjugation was achieved in either case. However, when using *L. cremoris* NZ9000 *traR*_*pNP40*_::Ter as donor in the non-optimal solid mating protocol, the conjugation frequency was 0.88%, in contrast to the 7.78 × 10^–3^% achieved in the control. In the optimized spread solid mating protocol, inactivation of *tra*R in the donor strain caused a twofold increase in conjugation frequency, while in the suboptimal solid mating protocol, its inactivation prompted a 110-fold increase in conjugation, compared to the unmodified *L. cremoris* NZ9000 pNP40 strain. Furthermore, when attempting conjugation under liquid mating conditions, which yielded no measurable conjugation in any of the previous cases, when using *L. cremoris* NZ9000 *traR*_*pNP40*_::Ter as a donor, a conjugation frequency of 4.83 × 10^–5^% was achieved. Although this conjugation frequency was the lowest obtained so far, it was significantly higher than the complete lack of conjugation observed for the control, where *L. cremoris* NZ9000 pNP40 was used as a donor, which further proves the increased conjugation phenotype displayed by *L. cremoris* NZ9000 *traR*_*pNP40*_::Ter. These results suggest that the product of gene *tra*R acts as a negative regulator of pNP40 conjugation and that, when inactivated, conjugation frequencies significantly (*P* < 0.05) increase. This stimulatory effect of inactivating *tra*R is better observed under suboptimal conditions for conjugation.

**TABLE 5:** Conjugation frequencies obtained when using different strains as donors, using three different mating protocols: non-optimal solid mating, liquid mating, and spread solid mating.

Lactococcal *s*train used as donor	Spread solid mating (%)	Non-optimised solid mating (%)	Liquid mating (%)
NZ9000 pNP40, pJP005	2.34	7.78 × 10^–3^	0
NZ9000 *tra20*_*pNP40*_::Ter	2.24	7.02 × 10^–3^	0
NZ9000 *tra19*_*pNP40*_::Ter	2.1	6.4 × 10^–3^	0
NZ9000 *traR*_*pNP40*_::Ter	4.8	0.88	4.83 × 10^–5^

*These strains are all *L. cremoris* NZ9000 harboring pNP40 and pJP005, in which three different conjugation-related genes from the pNP40 conjugation cluster were individually mutated in order to ascertain the effect that these individual mutations would have in the overall conjugation frequency. In all cases, *L. cremoris* MG1614 was used as the recipient strain.*

### pNP40-Mediated Plasmid Co-mobilization

The genomic content of pNP40-containing strain *L. lactis* subsp. *lactis* biovar diacetylactis DRC3 (chromosome size of 2,385.405 kb) was sequenced, and seven plasmids were identified to be present in this strain. These plasmids were named using letter assignations based on their respective sizes (largest to smallest): pDRC3-A (103.88 kb), pDRC3-B (64.98 kb, identified as pNP40), pDRC3-C (43.49 kb), pDRC3-D (8.27 kb), pDRC3-E (5.82 kb), pDRC3-F (3.35 kb), and pDRC3-G (2.66 kb).

Following the sequencing of the genomic content of *L. lactis* DRC3 and the identification of pNP40 in addition to six non-conjugative plasmids present in this strain, it was hypothesized that, during a conjugation event, pNP40 may be able to co-mobilize one or more of these plasmids into a recipient cell (such as *L. cremoris* MG1614). If pNP40-mediated co-mobilization were reported for *L. lactis* DRC3, definition and understanding of the specificity and rate at which this co-transfer of plasmids takes place and the identification of any common traits among the co-mobilized plasmids could be used to further exploit pNP40 as an agent in the mobilization of industrially relevant plasmids incapable of self-transfer. For this purpose, a multiplex PCR was designed incorporating eight pairs of primers that were specific to unique regions within each of the seven plasmids present in *L. lactis* DRC3 (and one region in its genome).

**FIGURE 4:**
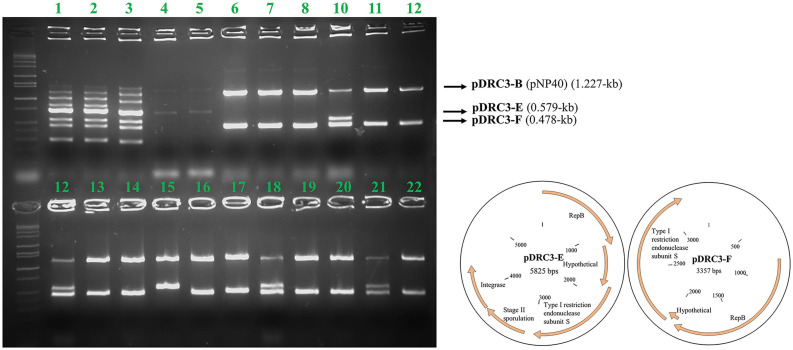
Agarose gel electrophoresis results using Multiplex PCR. For every lane, all eight primers designed for this purpose were used. Lanes 1–3 correspond to the donor (DRC3), for which PCR products representing all plasmids can be observed: pDRC3-A (1,483 bps product size), pDRC3-B (1,227 bps), pDRC3-C (1,000 bps), DRC3 chromosome (852 bps), pDRC3-D (723 bps), pDRC3-E (579 bps), pDRC3-F (478 bps), and pDRC3-G (306 bps). Lanes 4–5 correspond to the recipient (MG1614), while lanes 6–22 are representative of randomly selected transconjugants. Plasmid DRC3-B (pNP40) was transferred in all cases, whereas plasmid DRC3-E was co-transferred in ∼30% of the colonies screened and plasmid DRC3-F was co-transferred in all but one case. A plasmid map of the co-transferred plasmids is also presented.

Conjugation was performed using *L. lactis* DRC3 as a donor and *L. cremoris* MG1614 as a recipient, using the spread solid mating protocol, and 100 random colonies were screened from all resultant plates. As shown in Figure 4, and as a positive control for conjugation, in all the screened colonies plasmid pNP40 (pDRC3-B) was present, proving its successful transfer into the recipient cells. Two of the smaller plasmids, pDRC3-E and pDRC3-F, were co-mobilized by pNP40 at different frequencies. Plasmid pDRC3-E was co-mobilized in ∼30% of the screened colonies, whereas pDRC3-F was transferred along with pNP40 in 99% of the tested transconjugants.

## Discussion

Since conjugation is regarded as a food-grade process, it has been widely exploited to enhance starter cultures in the dairy industry (Mills et al., 2006). Despite the importance and application of conjugation, very little is known about its mode of action in lactococcal plasmids in comparison to other better-defined systems in Gram-positive bacteria, such as the *Enterococcus* model plasmids pIP501, and pCF10, or the *Bacillus* plasmid pLS20 (Goessweiner-Mohr et al., 2014; Kohler et al., 2019). Consequently, a genetic dissection of the pNP40 conjugation-associated operon was performed to obtain initial insights into this system as a model of lactococcal conjugation systems. Modifying previous protocols (Harrington and Hill, 1991; Trotter et al., 2001), the conjugation approach was optimized, achieving the best results employing the spread solid mating protocol. We hypothesize that the use of overnight cultures and the subsequent combination of both donor and recipient strains in a 1:1 ratio play a significant role in the improvement in conjugation frequency. Furthermore, the resuspension of the donor-recipient mixture in a relatively high volume of 5% RSM and 2% glucose and the even spread onto 5% RSM and 2% glucose agar plates seem to be the most crucial aspect for this conjugation improvement. When this cell mixture is spread rather than spotted on the center of the plate, we theorize that more nutrients are available to a bigger proportion of the cells, thus increasing the chance of mating.

Plasmid pNP40 was shown to be able to co-mobilize two of the six other plasmids present in *L. lactis* DRC3 at high frequency, which illustrates the potential of pNP40 to be used to mediate transfer of technologically desirable, non-self-transferable plasmids. Both mobilizable plasmids have a *rep*B gene in common, encoding a replication protein, and a type I restriction endonuclease subunit S gene. One possible explanation for this co-mobilization may be that these plasmids replicate via a rolling circle-type mechanism, which is also the way in which conjugation is believed to occur (Waters and Guiney, 1993). This may imply that RepB-associated ssDNA of these plasmids is allowed to be passed on to the conjugation apparatus at some point during the DNA transfer process. Another theory we present is that a similar *ori*T recognition sequence present in the co-transferred plasmids may be implicated in their co-mobilization (Francia et al., 2004).

**FIGURE 5:**
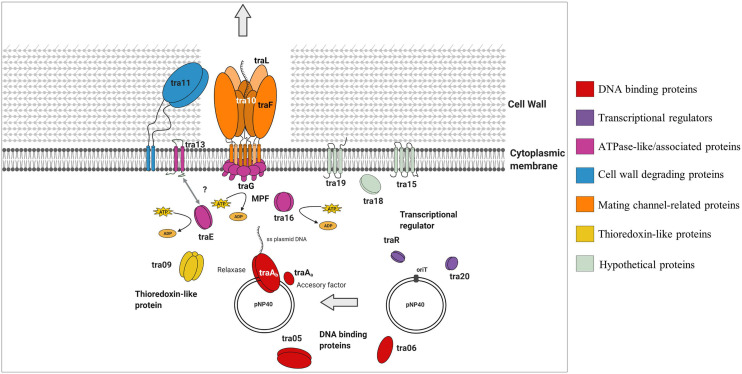
Schematic representation of the role that the different gene products from the pNP40 conjugation cluster play during the transfer of ssDNA in a conjugation event in a Gram-positive bacterium. This functional model is based on the mutational analysis of the eighteen genes that encompass this conjugation operon, paired with a predictive functional analysis based on sequence and structural similarities, although it should be emphasized that the function of many of these proteins requires experimental validation.

A predictive functional analysis of the pNP40 conjugation operon, paired with the results obtained from the mutagenesis of the eighteen genes that encompass this operon (and complementation and overexpression), has allowed us to create a functional model of the pNP40-mediated conjugation process (Figure 5). Conjugative transfer of pNP40 is initiated when the relaxase TraAb and its auxiliary factor TraAa introduce a single stranded break in the *nic*-site of the *ori*T and unwinds the super coiled DNA strand from the non-nicked strand. These genes would also be responsible for ssDNA recircularization once inside the recipient cell (Trokter and Waksman, 2018). We show that both these genes are essential for the conjugation process to occur. Following this cleavage, the ssDNA molecule is translocated to the recipient cell via a membrane-associated complex, belonging to the T4SS and a mating pair formation (MPF) system (Cabezón et al., 2015). A type IV coupling protein (T4CP) recruits the relaxosome (the relaxase-DNA complex formed after the relaxase nicks the double-stranded DNA) to the mating channel (Redzej et al., 2017). In pNP40-mediated conjugation, TraG and TraE (both essential for conjugation) are proposed to act as coupling proteins that recruit the relaxosome to the secretion channel in an ATPase-dependent manner. The ATPase-like Tra16 was also classified as essential for conjugation, which exemplifies the key role that all ATPase/ATPase-associated proteins play in the conjugation process of pNP40. Proteins Tra05 and Tra06, which were deemed to play a major role in conjugation, present DNA-binding motifs and could be part of the relaxosome or the machinery that recruits it to the secretion channel, although their specific role has yet to be determined.

The Tra13 protein, which was demonstrated to be essential for conjugation, is related to the PrgI family of proteins. Many of Gram-positive T4SSs have genes encoding small proteins with two or three predicted transmembrane domains directly upstream of the *vir*B4-like genes (Wallden et al., 2010). Such is the case of *prg*I in the pCF10 system, which overlaps the VirB4-like *prg*J, and it has been postulated that the two gene products may functionally interact together (Alvarez-Martinez and Christie, 2009). This is also the case for *tra*13, which is located directly upstream of, and overlapping with, the ATPase-encoding *tra*E, and encodes a protein presenting two predicted transmembrane domains, thus suggesting that Tra13 and TraE may be interacting together, although the details of this interaction are not yet understood. The non-perfect restoration of conjugation frequency obtained when complementing either *tra13* or *traE* may be due to a number of reasons, such as insufficient production of the gene product from the plasmid. Alternatively, since these genes are adjacent in the pNP40 conjugation cluster and may be translationally coupled, the mutation of one of them and subsequent expression from an unlinked expression vector may lead to a suboptimal Tra13:TraE ratio for conjugation.

Tra11 is suggested to act as the peptidoglycan hydrolase of this system, responsible of degrading the thick peptidoglycan wall present in Gram-positive bacteria, facilitating the donor-recipient cell fusion required for the transfer of ssDNA during conjugation, and its mutation was shown to have a major impact on conjugation frequency, highlighting the important role that cell wall degradation plays in the conjugation process of pNP40. Proteins Tra10, TraL, and TraF are hypothesized to form the actual mating channel in the donor cell envelope. TraL is essential for conjugation, thus being the minimal component that this mating channel requires. Mutation of TraF caused a major negative impact on conjugation, thus appearing to be an important, yet not absolutely essential, mating channel protein, whereas mutation of Tra10 had only a modest impact on conjugation, and its role in forming and maintaining the mating channel appears not to be required. The roles of Tra15 and Tra19 in the conjugation process of pNP40 are still uncertain, but they appear to not be essential for conjugation. Conversely, mutation of *tra18* was shown to completely inhibit conjugation, which demonstrates the key role that this gene plays in conjugation of pNP40, although its function in conjugation is yet uncharacterized. Tra09 is a thioredoxin-like protein, with a yet unclear role in conjugation, although its inactivation had only a minor impact in conjugation, thus deemed not essential for conjugation. Thioredoxin proteins can be found in some (large and frequently conjugative) plasmids, although their absence in several conjugative plasmids suggests that they are not generally required for maintenance or transfer of a conjugative plasmid, even though they may enhance stability of proteins spanning the periplasmic space (Hemmis and Schildbach, 2013).

Finally, both Tra20 and TraR display similarities to the MerR family of transcriptional regulators. The repressor of the Gram-positive broad host-range plasmid pIP501, TraN, also displays structural similarities to this family of regulators and its overexpression has been suggested to diminish horizontal gene transfer of pIP501 (Kohler et al., 2019). To ensure that conjugation is only activated under favorable conditions, many conjugation clusters are tightly regulated. Such regulation has been reported in the conjugative plasmid pLS20 from *B. subtilis*, in which the main conjugation promoter located upstream of the conjugation operon is repressed by Rco_*pLS20*_, which in turn is present in low levels to allow for a sensitive and rapid switch under favorable conditions for conjugation (Meijer et al., 2021). The precise function of Tra20 and/or TraR in the regulation of pNP40 conjugation will be subject of future investigations.

The mutational mapping of the *tra* operon of pNP40 achieved in this study has allowed the genetic delineation of the conjugation cluster of this and other plasmids with a related conjugation cluster, which are prevalent among the demonstrated lactococcal conjugative plasmids. The work described herein should therefore be considered as a springboard for further functional investigations.

## Data Availability Statement

The data presented in the study are deposited in the NCBI Genbank repository, accession numbers CP064835, CP064836, CP064837, CP064838, CP064839, CP064840, CP064841 and CP064842.

## Author Contributions

GO, AB, JM, and DS conceived and designed the experiments. GO and AB performed the experiments. PK was responsible for the genomic assembly and annotation. GO analyzed the data and wrote the first draft of the manuscript. GO, HS, ET, PW, JM, and DS discussed and analyzed the results, and commented on and edited the manuscript. All authors contributed to the final manuscript and approved the submitted version.

## Conflict of Interest

HS, ET, and PW were employed by the company DSM. The remaining authors declare that the research was conducted in the absence of any commercial or financial relationships that could be construed as a potential conflict of interest.
