# Supplementary material for: Genetic Dissection of a Prevalent Plasmid-Encoded Conjugation System in Lactococcus lactis
Source: Front Microbiol. 2021 May 28;12:680920. doi: 10.3389/fmicb.2021.680920 (PMC8194271; doi:10.3389/fmicb.2021.680920)
Supplement: Supplementary file 1 [file Table_2.DOCX]

**Supplementary Table S2.** Oligonucleotides used in this study. Sequences of oligonucleotides used to: insert stop codons via recombineering, PCR screen for the mutations, cloning of genes into pPTPi and pNZ44E, and of intergenic regions into pPTPL, PCR screen for these transformations, PCR screen for plasmid co-mobilisation in DRC3-mediated conjugation and PCR screen for pNP40 and MG1614.

| Oligonucleotide name | Oligonucleotide sequence (5’ → 3’) |
| --- | --- |
| Recombineering primers^ᴪ^ |  |
| Tra20_pNP40_::Ter | G*T*T*C*T*TTTTAATATAATTCAAACCTTCATCTGAAATTAATTACCGAATTCAAATTCGCCCATGTTTTTGAATCATTGTACTTTTAAATAG |
| Tra19_pNP40_::Ter | C*A*T*A*G*GTATCAATTTAGTTATCAACGATATAGCAATAGCTTAACGAATTCATATCAGAAGAATAAATGAATAAGGTATTAGTATTATCAA |
| Tra18_pNP40_::Ter | A*T*T*C*T*CTGATAGATTTTCATTTAAAACTTCACTTGCATTTTAAAGAATTCATATTGGTTTGCTAAGTTTTCTATTTTTATCATCTATAAC |
| TraG_pNP40_::Ter | A*G*A*T*T*TTTCTAAAAGGAAATAGTAAGTAAAATGATTGAATTAAAGAATTCATACACCTATGAAAATTGCCAAGATTGATAAAAGTATTAT |
| Tra16_pNP40_::Ter | A*A*C*A*A*TTCCAACAATGATTAAACCGATTGAGCCTGCACCTTAAAGAATTCAGAACCAAGTCAATAACTTCTTAATTCCGTCAAGGGCGGG |
| Tra15_pNP40_::Ter | G*G*T*G*T*CCTGATTGGAGAAATTGTTCTAGTTATTGCATGGTGAATTCTTTAATATTATTTAGGACACTTGGATAAGTTAAATGAAAAGCTG |
| TraL_pNP40_::Ter | T*A*G*G*A*AAAATATGATTAAATATGTTCCTACTTTAACAGTTTATGGAATTCAACTAGAAAGTACATCAGTTACATATTTCCAAGCATTAGT |
| Tra13_pNP40_::Ter | G*G*T*A*G*CTAAAAATATCATCAATAATGCTAAAAATTGTCTTTAGAGAATTCATGTCATGCCACAAACTTTTGCATGTATTTTTGAACCGTC |
| TraE_pNP40_::Ter | A*T*T*T*T*CAAAAAGAGAAGTGTATTTTAACGTATTTTGACTTTAAGGAATTCAAGTTTTTTTAGCTCGATTAGCATTAGTTTTCAAACGTTT |
| Tra11_pNP40_::Ter | A*C*A*A*G*AGTTGCCAATAAAGCTAAACAGATAAGCAACCCTTGAATTCATTAAGTTGTTGGTTTTTCAGAAATAAACGAAAAAGATAATAGT |
| Tra10_pNP40_::Ter | A*A*A*T*T*TTTTAAAATATTAAGTGGTGTTTTCGGAGTAGCATGAATTCTGTAACTTATTGCAGTAGTAGCTCTGTTTGCGACAAGCATTAGT |
| Tra09_pNP40_::Ter | A*T*A*G*A*TAAAGACAAAATACAATTATTCTATCGTGATGACTGAATTCGTTAACCGGACTGTAAAAAGATATTCAAACAAATTTATCTACAC |
| TraR_pNP40_::Ter | A*A*A*G*A*AGTTAAAAAGAAAATCGAGGATAGCGAGAAAGACTGAATTCATTAACTTATGGAATTTCTAAAAAATTCTAAAATGACAATTCAT |
| TraF_pNP40_::Ter | G*A*A*T*A*CATTGATTTAATTAAGTTAGTTATTGATACAAACTGAATTCCTTAAGAATTGTTGAAATTATATGAACTAAACACAAAGTCAGTT |
| Tra06_pNP40_::Ter | A*A*T*T*C*AGAAATGTTTAAAAACTATCTAAAATTTGTTGCTTGAATTCCTTAATGCCCTCACTATTCTTCAAGAAATTTGAGATTTTTACAA |
| Tra05_pNP40_::Ter | A*A*T*A*A*ATTTCCAACTCCAAAAAAAGTATTGATGGAGATGTGAATTCTATAAGAAGAAATGATACAGGAACAATTAGATTTAATTGATAAA |
| TraA_a-pNP40_::Ter | A*C*A*G*G*TATCAATAATTTTTCTATTTTTGCTAGACGAGCTTGAATTCGTTAATGCAATAAAGAGATTTTTACTCTTGATTTTTCAGAATAT |
| TraA_b-pNP40_::Ter | T*G*T*A*A*AACCATGAAAGAATATCTTGGAAATTCTGCGGAGTGAATTCTTTAAATTATCGCCACTCATACTGATAAAGACCACCTGCATAAT |

| Recombineering screening primers |  |
| --- | --- |
| ScrnTra20-Fw | AAACCAGTTCACAACGATTAAG |
| ScrnTra20-MAMAFw | GGGCGAATTTGAATTCGGTAA |
| ScrnTra20-Rv | TTGTATCCTCCTTGTTTCCTAA |
| ScrnTra19-Fw | AGATGTTGTTACCTTGTAATTCTG |
| ScrnTra19-MAMAFw | CTGATATGAATTCGTTAAGCTATTG |
| ScrnTra19-Rv | CTTTGATAGCCTCTTTATTCTCTG |
| ScrnTra18-Fw | TTATTCTTCTGATATGTGCTATTG |
| ScrnTra18-MAMAFw | CAAACCAATATGAATTCTTTAAAATG |
| ScrnTra18-Rv | CCAGTAAGAATAGTAATAAAGGATAG |
| ScrnTraG-Fw | ATGATGAAGAAGCGGTTAG |
| ScrnTraG-MAMAFw | GGTGTATGAATTCTTTAATTCAATC |
| ScrnTraG-Rv | TCATGTAGTAACTTCCCTTTAG |
| ScrnTra16-Fw | ATATAAAGACGACTGGCAAAG |
| ScrnTra16-MAMAFw | CTTGGTTCTGAATTCTTTAAGG |
| ScrnTra16-Rv | ACAACAACACCAATAAGCAAAG |
| ScrnTra15-Fw | AAAATTTAGTACAAGCTCACTC |
| ScrnTra15-MAMAFw | TTGCATGGTGAATTCTTTAATA |
| ScrnTra15-Rv | GGTAACTCCTGCTTGTAG |
| ScrnTraL-Fw | AGGGTGTCCTGATTGGAG |
| ScrnTraL-MAMAFw | ACTTTCTAGTTGAATTCCATAAAC |
| ScrnTraL-Rv | CAAAGCCTTGCAAACATACC |
| ScrnTra13-Fw | AATGTAATTTCTCCGTCAAGTC |
| ScrnTra13-MAMAFw | GCATGACATGAATTCTCTAAAG |
| ScrnTra13-Rv | GTTCCTCGTCAGAAATAGTG |
| ScrnTraE-Fw | CGTCAGCACCGTCAATTTGG |
| ScrnTraE-MAMAFw | AAAAAAACTTGAATTCCTTAAAGTC |
| ScrnTraE-Rv | AATCGTTCGCCAACTTCCAC |
| ScrnTra11-Fw | AAGGGTAGTGGTCTAATTATTG |
| ScrnTra11-MAMAFw | AGCAACCCTTGAATTCATTAAG |
| ScrnTra11-Rv | GAAGTACCACTAGGGTTTGT |
| ScrnTra10-Fw | GCCTAATGTAATACCAAACAATG |
| ScrnTra10-MAMAFw | GAGTAGCATGAATTCTGTAAC |
| ScrnTra10-Rv | TTCTCCTAACACATGGAAATC |
| ScrnTra09-Fw | GCAGGTCAAGGCAAAGTAG |
| ScrnTra09-MAMAFw | GTGATGACTGAATTCGTTAACC |
| ScrnTra09-Rv | GATAGCTTCCGCTTGGTC |
| ScrnTraR-Fw | CAACCCACCAAAGATAATTTAC |
| ScrnTraR-MAMAFw | GAAAGACTGAATTCATTAACTTATG |
| ScrnTraR-Rv | AGCCTTGTCATTGATTACCG |
| ScrnTraF-Fw | AGATATGCAGGTACTCAAAAAG |
| ScrnTraF-MAMAFw | TGATACAAACTGAATTCCTTAAGA |
| ScrnTraF-Rv | TTTATCGGCTTTTAGTGCATTC |
| ScrnTra06-Fw | ATCACCAGACAAAGGCAAAC |
| ScrnTra06-MAMAFw | TGTTGCTTGAATTCCTTAATGC |
| ScrnTra06-Rv | CGACAAAAGCGGTAGATTC |
| ScrnTra05-Fw | GCACAAAGAGAGTCAAGAAG |
| ScrnTra05-MAMAFw | GATGGAGATGTGAATTCTATAAG |
| ScrnTra05-Rv | CAACAAGCTCGTCTAGCAAA |
| ScrnTraA_a_-Fw | AGAAGCCATTCAACTGACAAATA |
| ScrnTraA_a_-MAMAFw | CGAGCTTGAATTCGTTAATGC |
| ScrnTraA_a_-Rv | CTCCGCAGAATTTCCAAGATA |
| ScrnTraA_b_-Fw | CTTCGACAAAATCTGAACTCAAACGTA |
| ScrnTraA_b_-MAMAFw | GCGGAGTGAATTCTTTAAATTATC |
| ScrnTraA_b_-Rv | TCAACACAGCGTGCAGGTATCTTAATC |
|  |  |
| Complementation primers^₳^ |  |
| pPTPi-Tra18-Fw | AAAAAAGTCGACAGGAGGCACTCACCATGAATGATGAAGAAGCGGTTA |
| pPTPi-Tra18-Rv | AAAAAAGAATTCTTACATTTCTAAGTCCATTCCTTT |
| pPTPi-TraG-Fw | AAAAAAGTCGACAGGAGGCACTCACCATGGTTAAAAAAACAAAAATAATACT |
| pPTPi-TraG-Rv | AAAAAAGAATTCTTAGTCATTAAATTGGCTATAAATA |
| pPTPi-Tra16-Fw | AAAAAAGTCGACAGGAGAAAATAATTATGAATAAACTTG |
| pPTPi-Tra16-Rv | AAAAAAGAATTCTTAAGCTGTCGGAGGTGC |
| pPTPi-Tra15-Fw | AAAAAAGTCGACAGGAGGCACTCACCATGGAAAACTTGATAGAAGACA |
| pPTPi-Tra15-Rv | AAAAAAGAATTCACCTCCTAATTTCTTTTGCTAA |
| pPTPi-TraL-Fw | AAAAAAGTCGACAGGAGGTTCATTTTATGGACTT |
| pPTPi-TraL-Rv | AAAAAAGAATTCTTAACCTCCTAAACCAACGAC |
| pPTPi-Tra13-Fw | AAAAAAGTCGACAGGAGGCACTCACCATGAATTTTAATGTACGAGTATATAA |
| pPTPi-Tra13-Rv | AAAAAAGAATTCTTAGATAAAGGGGTCTGTTTC |
| pPTPi-TraE-Fw | AAAAAAGTCGACAGGAGGCACTCACCATGAGCATAAAGAATTTATTCAATC |
| pPTPi-TraE-Rv | AAAAAAGAATTCTTAGTCCGTTTGCATAATCTTA |
| pPTPi-Tra11-Fw | AAAAAAGTCGACAGGAGGCACTCACCATGGCAGGAAAGTATATTTTTAAG |
| pPTPi-Tra11-Rv | AAAAAAGAATTCATTGTTTGGTATTACATTAGGC |
| pPTPi-Tra10-Fw | AAAAAAGTCGACAGGAGGCACTCACCATGGAAAATAAATTTTTTAAAATATTAAGT |
| pPTPi-Tra10-Rv | AAAAAAGAATTCTTAATTATTTTTTGTATCAAGTTTAGTT |
| pPTPi-Tra09-Fw | AAAAAAGTCGACAGGAGGCACTCACCGTGATAGCCCTCACAGG |
| pPTPi-Tra09-Rv | AAAAAAGAATTCTCATTTTAAAATCTCCTTTATTTTCT |
| pPTPi-TraF-Fw | AAAAAAGTCGACAGGAGGCACTCACCATGAATTTAGGGACAAAAAAAAGT |
| pPTPi-TraF-Rv | AAAAAAGAATTCTAACTTTTTCATCATGAATTTTTATTT |
| pPTPi-Tra06-Fw | AAAAAAGTCGACAGGAGGCACTCACCATGGTTATTGATTTAGAAAAAGC |
| pPTPi-Tra06-Rv | AAAAAAGAATTCTCATAATTCCTTTTCTCCTTTG |
| pPTPi-Tra05-Fw | AAAAAAGTCGACAGGAGGCACTCACCATGATTAACTATCAAGGCGAAG |
| pPTPi-Tra05-Rv | AAAAAAGAATTCTTAATTCTCATAATTTATATTTTTTGGAA |
| pPTPi-TraA_a_-Fw | AAAAAAGTCGACAGGAGGCACTCACCATGAAAAAAATCAAAAATCGTGAAA |
| pPTPi-TraA_a_-Rv | AAAAAAGAATTCTTACTCCCCCTCTGAAATAA |
| pPTPi-TraA_b_-Fw | AAAAAAGTCGACAGGAGGCACTCACCATGACAGTCATTAAAATGCCAAA |
| pPTPi-TraA_b_-Rv | AAAAAAGAGCTCCTAAATTTCAAAGTCATCATCAC |
| ScrnpPTPi-Fw | TGATTTCGTTCGAAGGAACTA |
| ScrnpPTPi-Rv | TGGCGGACAATAAGTCCTC |
|  |  |
| Overexpression primers^₳^ |  |
| pNZ44-Tra20-Fw | AAAAAACCATGGAGGAGGCACTCACCTTGAAAGAAAATAATTGGTATACATT |
| pNZ44-Tra20-Rv | AAAAAAGAGCTCCTATTAGCCTTAAAAAATGTTAAGA |
| pNZ44-Tra19-Fw | AAAAAACCATGGAGGAGGCACTCACCATGCCAAATATACTATGGTATATG |
| pNZ44-Tra19-Rv | AAAAAAGAGCTCTCATTTTGTATCCTCCTTGTTT |
| pNZ44-TraR-Fw | AAAAAACCATGGAGGAGGCACTCACCATGAATAACCAAACCTTACAAG |
| pNZ44-TraR-Rv | AAAAAAGAGCTCCTAAAGCTAGTTACTGATAATGAT |
| ScrnpNZ44-Fw | GAGACGGGACGATAGCAAT |
| ScrnpNZ44-Rv | CTATCGAAAGCGAAATCAAACG |
|  |  |
| Co-mobilisation screening primers |  |
| ScrnDRC3A-Fw | AACATCTATCTTCGTGCTATTC |
| ScrnDRC3A-Rv | CAAACTCTGGCAACATTTCAC |
| ScrnDRC3B-Fw | ACATTAGCCGCTGTTTCC |
| ScrnDRC3B-Rv | TAGCCAAGATTGAGGTTCAC |
| ScrnDRC3C-Fw | CAGTTGCAGTCGTATTTCC |
| ScrnDRC3C-Rv | CGCTTTGGCATTTGATTAGG |
| ScrnDRC3D-Fw | TGCCACTTTCTGGTATCTATG |
| ScrnDRC3D-Rv | TTCCAGCAACAACCAGAATC |
| ScrnDRC3E-Fw | AAAGGAGTAAGCAAATGAATAATG |
| ScrnDRC3E-Rv | TAATCCAATCCGCAACATAAG |
| ScrnDRC3F-Fw | GTCAGTGTTTATAGGGAGTTAC |
| ScrnDRC3F-Rv | GTTTAAAGCGACTATGCTTATC |
| ScrnDRC3G-Fw | GCAGCAATGTATTCAAAAAATG |
| ScrnDRC3G-Rv | TGTCCAATGATTAGGTTGTAATTC |
| ScrnDRC3-Fw | CTTATCTGGTTTGGCCTATG |
| ScrnDRC3-Rv | ACAGCTTCTGGTAAAATTAACG |
|  |  |
| Conjugation screening primers |  |
| ScrnpNP40-Fw | TGTGGGAGAAGCTACTGGAGAATGGATACC |
| ScrnpNP40-Rv | TTGCTCGGACTGCCGGAATTGGTG |
| ScrnMG1614-Fw | AATGGCTCGGAGATGAAGAC |
| ScrnMG1614-Rv | ACAATTCCACGAAGGCTCTC |

* Phosphorothioate linkages of recombineering oligos.

ᴪ Introduced restriction enzyme sites (EcoRI) are underlined.

₳ Restriction enzymes sites are single underlined, while Shine-Dalgarno sequences from pNZ8048 are double underlined.
