# Supplementary material for: Genetic Dissection of a Prevalent Plasmid-Encoded Conjugation System in Lactococcus lactis
Source: Front Microbiol. 2021 May 28;12:680920. doi: 10.3389/fmicb.2021.680920 (PMC8194271; doi:10.3389/fmicb.2021.680920)
Supplement: Supplementary file 2 [file Data_Sheet_1.docx]

Supplementary Material

**Supplementary Figure S1.** Schematic representation of the three strategies taken in this study for the conjugation of the pNP40 plasmid between a donor strain and a recipient: (i) Solid mating, (ii) filter mating and (iii) spread solid mating.


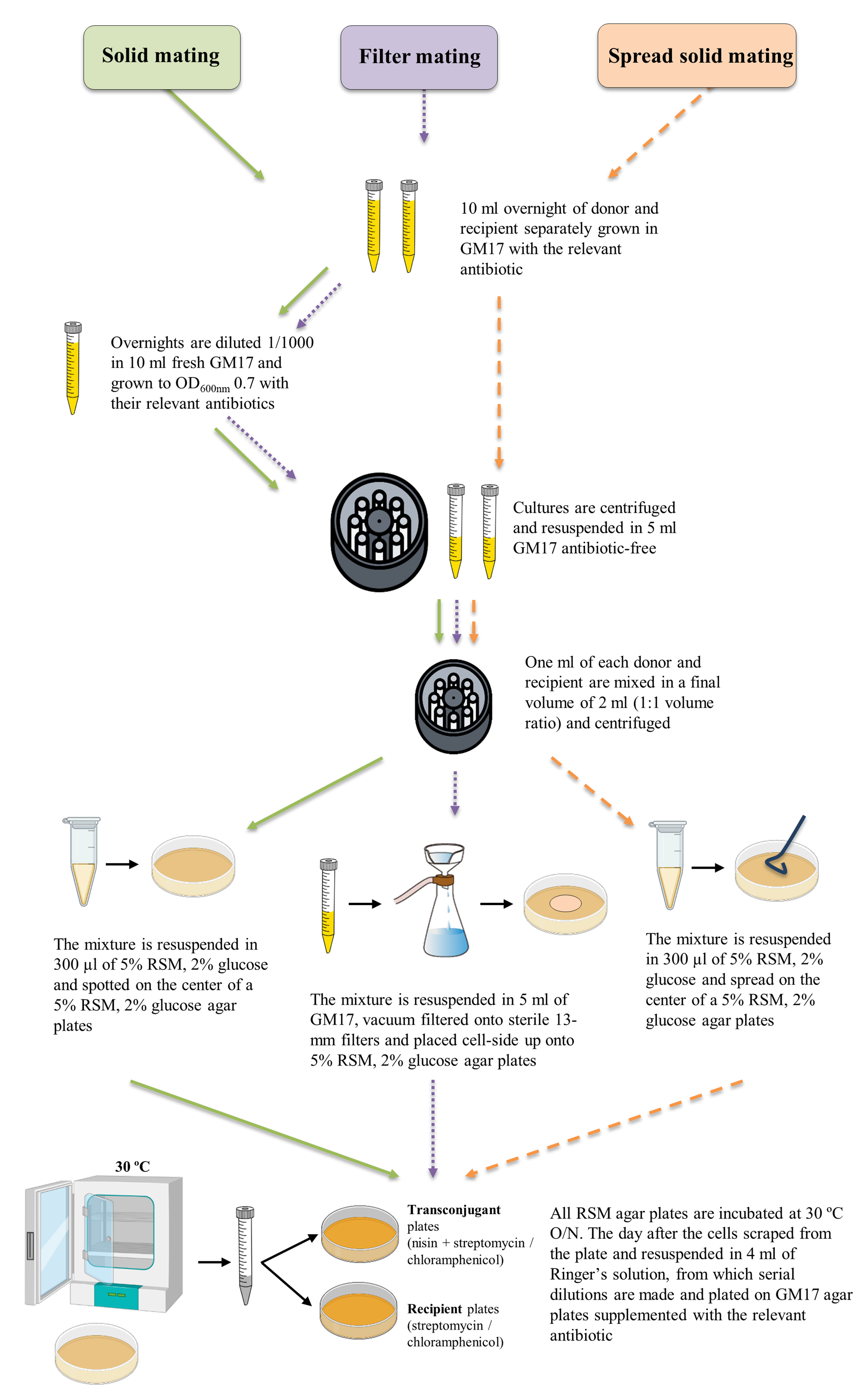


**Supplementary Table S3.** Sizes, in base pairs (bps), of the PCR products obtained using specific primers for the chromosome and each plasmid from *L. lactis* DRC3 for the co-mobilisation assay.

| **DRC3 genomic content** | **PCR product size (bps)** |
| --- | --- |
| pDRC3-A | 1,483 |
| pDRC3-B | 1,227 |
| pDRC3-C | 1,000 |
| DRC3 chromosome | 852 |
| pDRC3-D | 723 |
| pDRC3-E | 579 |
| pDRC3-F | 478 |
| pDRC3-G | 306 |

**Supplementary Figure S4.** Neighbor-joining tree of the 15 pNP40-like mobilisable plasmids. MEGA X was used for alignment using ClustalW and subsequent construction of the Neighbor-joining tree.


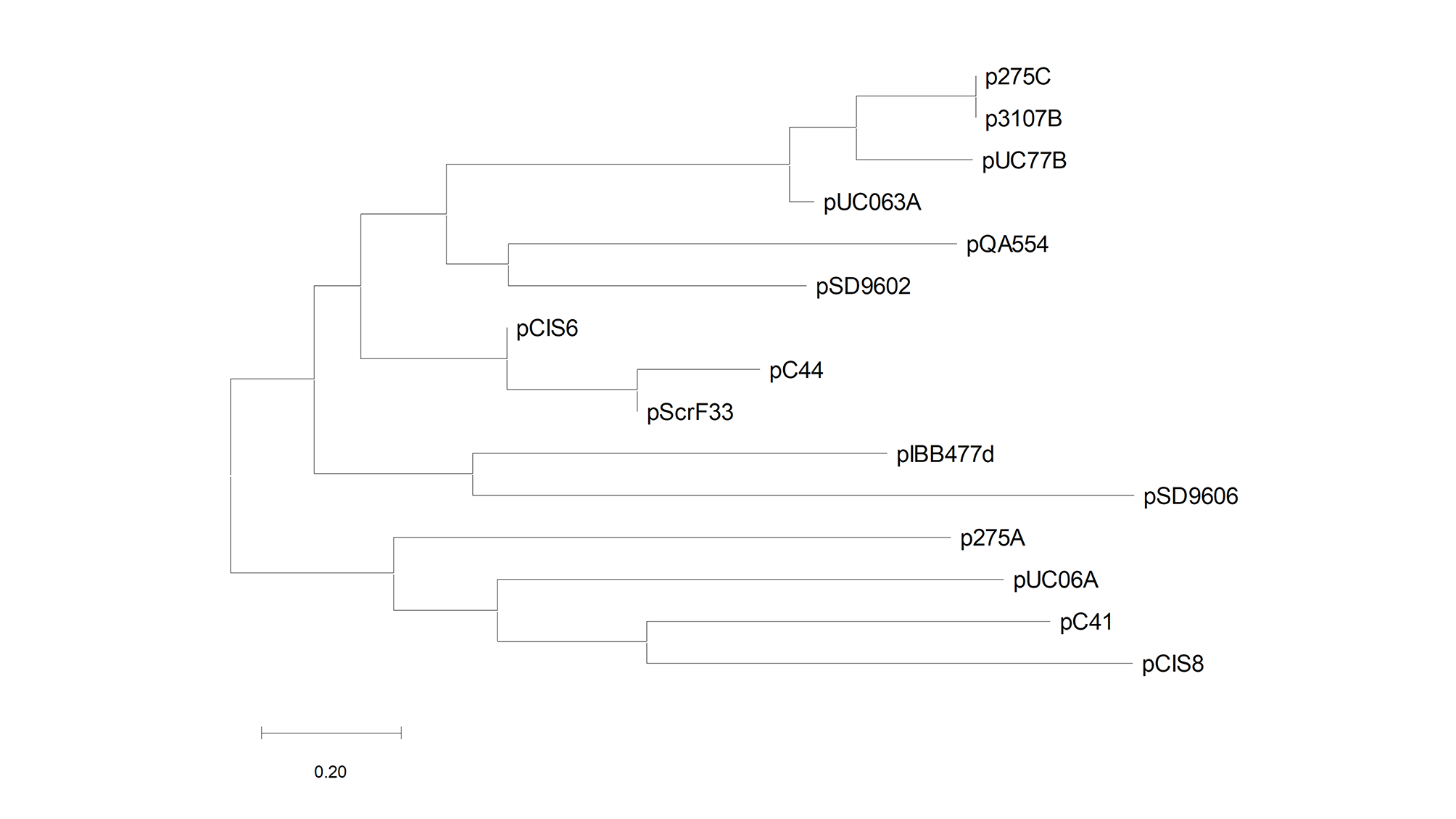


**Supplementary Table S5.** Conjugation frequencies comparison of the pNP40 plasmid among different studies and methods. Frequencies in previous studies are based on the number transconjugants per donor cell, which were multiplied by 100 in order to give a percentage, while frequencies in this study are based on the percentage of transconjugants per recipient cell.

| **Conjugation method** | **Conjugation frequency (%)** | **Source** |
| --- | --- | --- |
| **Solid mating (RSM)** | 3x10^-4^ | Harrington and Hill, 1991 |
| **Solid mating (GM17)** | 5x10^-4^ | Trotter *et al*., 2001 |
| **Solid mating (RSM, non-optimized)** | 8x10^-3^ | This study |
| **(i) Solid mating (OD_600nm_ 0.7)** | 0.125 | This study |
| **(ii) Filter mating** | 0.447 | This study |
| **(iii) Spread solid mating** | 1.686 | This study |
